# Syndrome of Reduced Sensitivity to Thyroid Hormones: Two Case Reports and a Literature Review

**DOI:** 10.1155/2016/7546453

**Published:** 2016-09-28

**Authors:** Anastasios Anyfantakis, Dimitrios Anyfantakis, Irene Vourliotaki

**Affiliations:** ^1^Department of Endocrinology, Venizeleio General Hospital, Heraklion, Crete, Greece; ^2^Primary Health Care Centre of Kissamos, Chania, Crete, Greece

## Abstract

Resistance to thyroid hormone (RTH) is an extremely rare dominantly inherited condition of impaired tissue responsiveness to thyroid hormone (TH). Most patients with RTH have mutations in the gene that encodes the *β* isoform of the receptor of thyroid hormone (*THR-β* gene). Mutant receptors are unable to activate or repress target genes. The majority of them are asymptomatic or rarely have hypo- or hyperthyroidism. RTH is suspected by the finding of persistent elevation of serum levels of free T3 (FT3) and free T4 (FT4) and nonsuppressed TSH. We present two cases of RTH diagnosed after total thyroidectomy. The first patient was initially diagnosed with primary hyperthyroidism due to toxic multinodular goiter. The second patient had undergone thyroidectomy for multinodular goiter 16 years before diagnosis of RTH. After thyroidectomy, although on relatively high doses of levothyroxine, both of them presented with the laboratory findings of RTH. Genetic analysis revealed RTH.

## 1. Introduction

RTH is an unusual dominantly inherited condition of impaired tissue responsiveness to TH, expressed clinically by the persistent elevation of serum levels of FT3 and FT4 and inappropriate high levels of TSH. These hormone levels reflect a compensated endocrine state in which increased levels of TH are required to establish normal levels of TSH. Despite the high TH levels, TSH responds to stimulation with TSH releasing hormone (TRH) and the levels of TH required to suppress TSH and produce metabolic effects on peripheral tissues are higher than normal [1]. The incidence of RTH is probably 1 case per 50000 live births [2]. Syndrome of RTH is rarely suspected due to its heterogenous presentation and atypical symptoms at onset. Goiter is the principal clinical finding which is usually further investigated [3]. In most cases the elevated TH levels compensate for the tissue resistance, so the individuals are euthyroid. However, not rarely, the compensation appears to be incomplete and hypothyroidism is produced. Rarely, high endogenous thyroid hormone levels can produce toxic effects on peripheral tissues. Here we present two cases with RTH. A literature review on the diagnosis and management of the condition is also performed.

## 2. Case Presentation

### 2.1. 1st Patient

A 58-year-old male was referred by his general practitioner to the Endocrinology Department of the Venizeleio General Hospital of Heraklion, Crete, Greece, due to frequent episodes of sinoatrial tachycardia. His personal medical history was free, with no known thyroid disease in his family. His weight and height were 79 kg and 175 cm, respectively (BMI 26). Clinically, his thyroid was enlarged with multinodular consistency at palpation. Laboratory investigation disclosed suppressed TSH with FT3 and FT4 levels higher than the upper normal limits (Table 1). AntiTg and antiTPO autoantibodies were negative. Thyroid ultrasound revealed multinodular goiter with a dominant nodule of 1.8 cm maximum diameter. Increased uptake was found on the Tc99 thyroid scan. Whole blood count, erythrocyte sedimentation rate, liver and renal function, and the rest of biochemical parameters were all normal, except for triglycerides, which were found elevated (230 mg/dL). The patient was diagnosed to have multinodular toxic goiter and was prescribed carbimazole at a starting dose of 45 mg/d, which was gradually tapered. Three months later, while on carbimazole 15 mg/d, he became euthyroid (TSH, FT3 normal), although FT4 was marginally elevated. This finding was not considered at that time. It should be mentioned that, after 3 months, while he was on carbimazole 15 mg/d, high levels of FT3 and FT4 did not suppress TSH, as expected; on the contrary, TSH was found to be a little elevated (Table 1). This finding was not considered at that time. Five months later he underwent near total thyroidectomy. Biopsy showed multinodular goiter with no signs of malignancy. There were no complications after surgery but it proved difficult to treat his postsurgical hypothyroidism, with levothyroxine at doses higher than usual.

While he was on levothyroxine 300 *μ*g/d, TSH remained elevated with FT4 and FT3 above the upper normal limits (Table 2). Differential diagnosis included either a TSH-producing pituitary adenoma or RTH, in which tissue insensitivity to the action of TH is compensated by elevated levels of these hormones without suppression of TSH. In order to discriminate between these two conditions, the patient had a TRH test while on levothyroxine 300 *μ*g/d. TSH increased from basal levels of 14.3 *μ*UI/mL to peak levels of 70 *μ*UI/mL thirty minutes after the i.v. administration of 200 *μ*g TRH.

The diagnosis of RTH was even more suspected, as MRI of the pituitary was negative for any lesion and the rest of the pituitary hormones were found normal. Next diagnostic test was genetic analysis for RTH, which proved to be positive. In terms of further investigation of RTH, metabolic markers affected by TH, such as cholesterol and triglycerides, were found elevated. Ferritin and liver enzymes were normal and Sex Hormone Binding Globulin (SHBG) was low normal (Table 3). Finally, the dose of levothyroxine was increased to 325 *μ*g/d. It was well tolerated and the patient presented with tissue euthyroidism, as it was evidenced by metabolic markers (Table 3). TH profile was the optimum until that time (Table 2).

### 2.2. 2nd Patient

A 32-year-old woman was referred by her general practitioner to our Department for post thyroidectomy hypothyroidism, resistant to usual doses of levothyroxine. She had had near total thyroidectomy at the age of 16 because of multinodular goiter with a dominant cold nodule. No malignancy was found on biopsy. Clinical examination was normal. Her weight was 70 kg and her height was 160 cm (BMI 28). Her personal medical history was free and no thyroid disease was reported in her family. At the time of referral and while on levothyroxine 150 *μ*g/d, her TSH was high, with FT3 and FT4 unexpectedly normal (Table 2). Liver and renal function tests were normal (Table 3). Thyroid ultrasound showed a small remnant with no nodules. The dose of levothyroxine was increased to 200 and then to 250 *μ*g/d, but TSH levels remained high, despite the fact that FT3 and FT4 were above the upper limits of the normal range (Table 2). Taking levothyroxine 250 *μ*g/d the patient complained of palpitations and at that time she did not try any higher dose. As in the first patient, in order to clarify the possibility of RTH, we performed a TRH test, while the patient was on levothyroxine 250 *μ*g/d. Her serum TSH increased from basal levels of 11 *μ*UI/mL to peak levels of 110 *μ*UI/mL thirty minutes after the i.v. administration of 200 *μ*g TRH. Consequently, the diagnosis of RTH was highly suspected. MRI of the pituitary did not show any lesion and the rest of the pituitary hormones were found normal.

Metabolic markers, such as cholesterol and triglycerides, were normal, ferritin was low normal, and SHBG was found low. Liver enzymes were within normal limits, except for Alkaline Phosphatase (ALP), which was high (Table 3). Genetic analysis revealed RTH syndrome. Finally, on levothyroxine 300 *μ*g/d the patient's TSH was decreased to just above the upper normal limits, FT3 and FT4 were marginally elevated, and metabolic markers remained within normal limits (Tables 2 and 3).

Concomitant administration of atenolol 25 mg/d controlled tachycardia.

## 3. Genetic Analysis

Genetic analysis was performed in both index cases by using high-molecular-weight DNA isolated from white blood cells [4]. Exons 7, 8, 9, and 10 of the* THRβ* gene were amplified by PCR using specific primers [5]. For sequencing, unwanted dNTPs and primers were removed from PCR products using ExoSAP-IT (# 78201, USB, Cleveland, USA). In brief, 2 *μ*L of ExoSAP-IT was added to 5 *μ*L of PCR product and was incubated at 37°C for 20 min and 80°C for 10 min. After that, we added 0.5 *μ*L (20 pmol) of specific primer and 2 *μ*L of sequencing Mix (BigDye® Terminator v3.1 cycle sequencing kit # 4336911, Applied Biosystems, Foster City, CA). Reactions were run in a 3100 automated DNA sequencer (Applied Biosystems, Foster City, CA). Sequence analysis of exon 10 in the 1st patient showed a heterozygous single base pair substitution of C to T at codon 383 resulting in a change from arginine to cytosine (R383C). This mutation has been previously described [6]. Sequence analysis of exon 10 in the 2nd patient showed single base pair substitution of C to T at codon 448 to the end (codon 461) increasing the polypeptide chain length by two aminoacids. This mutation has also been described previously [7].

## 4. Discussion

Both of our patients were found to have mutations on the exon 10 of the* THRβ* gene.

Exons 8–10 in the carboxyterminus of the* THRβ* gene are the most common spots of mutations described in RTH [8]. Codon 453 is the most frequent site of mutations. RTH is generally inherited in the autosomal dominant manner. A total of about 150 different* THRβ* gene mutations have been identified so far [9, 10]. No mutation has been found to date in the* THRα* gene. Thyroid hormone receptors (THRs) are encoded by two genes (*THRα*,* THRβ*) located on chromosomes 17 and 3, respectively. There are 4* THR* isoforms:* THRβ1* and* THRβ2* derived from the* THRβ* gene and* THRα1* and* THRα2* from the* THRa* gene. They have in common a DNA binding domain (DBD) at their aminoterminus and, with the exception of the* THRα2* isoform, a ΤH (ligand) binding domain (LBD) at their carboxyterminus. The THRs associate with specific DNA sequences termed thyroid response elements (TREs). The THRs associated with TREs usually become functional only after binding of T3 on LBD. This produces a modulation in the rate of transcription of the target gene [11]. In the absence of T3, THR homodimers and heterodimers are associated with corepressors (NCoR and SMRT) that repress or silence the transcription of genes positively regulated by the ligand.

Binding of T3 to THRs releases the corepressors and recruits nuclear coactivators which stimulate gene transcription. Mutant THR*β*s interfere with the function of the wild type THRs, a phenomenon termed dominant negative effect (DNE). The DNE involves the occupation of a TRE by a mutant THR that cannot bind T3 or has reduced affinity for it,* tighter affinity* for the corepressors, or reduced ability to recruit coactivators necessary to enhance gene transcription. In some patients, mutations in the LBD are reported to maintain TH binding and yet cause RTH in certain tissues, due probably to selective impairment of TH-mediated gene repression [12].

Mutations in the* THRβ* gene were not found in a subgroup of patients with RTH. In the absence of* THRβ* mutations, an RTH phenotype could be caused by abnormal corepressors that fail to dissociate from THRs or defective coactivators that do not associate with THRs upon T3 binding [13–16]. There have also been reported cases of mosaicism, with the* THRβ* mutation present in some cell lineages, but not in others [17]. Most RTH patients undergo thyroidectomy for multinodular goiters. Goiter is a very common finding in RTH and is attributed to the continuously high levels of TSH. Additionally, in this syndrome TSH is reported to be of enhanced bioactivity. This could explain the relatively high percentage of RTH patients with goiter in the presence of normal levels of TSH and elevated TH levels [18].

There are no pathognomonic symptoms associated with RTH.

Its presentation is heterogenous [19]. The majority of individuals are completely asymptomatic, achieving normal growth and mental development, because the elevated thyroid hormone levels compensate for the tissue resistance.

In asymptomatic patients, Pulcrano et al., although, report the presence of echocardiographic signs similar to those reported in hypothyroid patients [20].

Diagnosis of the syndrome in asymptomatic patients is usually made on routine laboratory investigation in which “inappropriately high” TSH is discovered. In contrast, some patients with TH levels not necessarily elevated appear to be hypermetabolic with rapid heart rate [21]. More rarely, when RTH is not compensated, patients may present hypothyroid, especially in the rare cases where autoimmune thyroiditis coexists [22–24].

A possible reason for the variability in symptoms could be that not all the individuals express the same levels of normal and mutant THRs in their tissues [25]. Furthermore, not all mutations have the same effect on T3 binding [26]. Another factor could be the different tissue distribution of THRs' isoforms. For instance the heart is a predominantly THR*α* tissue. As the THR*α* is normal in RTH patients but their FT3 levels are high, it can be expected that they will react to the extra amount of T3 in a hyperthyroid manner, as far as heart is concerned [27]. Furthermore, not every individual will express the same amount of THRs or corepressors/coactivators in a particular tissue, leading to differences between patients.

For the diagnosis to be confirmed, the demonstration of reduced sensitivity of peripheral tissues to endogenous or exogenous TH is required. The degree of central resistance is assessed by the response of TSH to the TRH stimulation test. Usually, in RTH the TSH response to TRH is either normal or slightly exaggerated. In both of our patients, TSH showed a rather exaggerated response to TRH, while they received relatively high doses of levothyroxine. This is in accordance with RTH and also ameliorates the possibility of a TSH secreting pituitary adenoma. The majority of such adenomas are characterized by autonomous secretion of TSH [28], which neither responds to TRH nor is suppressed by increasing doses of administered T3 or T4. Peripheral resistance is estimated by metabolic markers such as serum SGOT, SGPT, ferritin, SHBG, cholesterol, and triglycerides. They are measured to determine the effect of TH on metabolism and hepatic function. CK and ankle jerk relaxation time are assessed to determine the effect of TH on neuromuscular system. Basal cardiac status is assessed with an echocardiogram and heart rate measurement.

Administration of TH in individuals without RTH causes alteration of these indexes, demonstrating a sensitivity to the rising T3 levels. In contrast, patients with RTH respond only to high doses of levothyroxine. The degree of sensitivity varies among tissues. Heart rate alone is a poor indicator of RTH status [29]. Such tests lack sufficient specificity and sensitivity to discriminate normal subjects from individuals with RTH. Lack of specificity is due to genetic and dietary factors, as well as alterations produced by age and sex. For these reasons the value of tests estimating the effects of TH on peripheral tissues is enhanced if determinations are obtained before and after the administration of TH with the subject serving as its own control [29]. In most patients, RTH appears to be adequately compensated (TSH near normal) by the increased endogenous supply of thyroid hormone. The 2nd patient seemed to belong to this category, as she was eumetabolic before thyroidectomy. Treatment should not be given to such individuals. Treatment with TH is reserved for those who, due to misdiagnosis, have received ablative therapy and have limited thyroid reserve and for subjects in whom the compensation appears to be incomplete due to the concomitant presence of autoimmune thyroid disease [1].

When RTH is not compensated, thyroid hormone should be given in incremental doses and parameters that are directly linked to TH action should be followed. Large goiters, a relatively common finding in RTH, are reported to regress with supraphysiological doses of TH [3]. In this direction as shown in Table 3, our patients did not become hypercatabolic, despite being treated with high doses of levothyroxine, which proved enough to bring TSH down almost to normal. The presence of tachycardia should not be a reason to detract treatment. It is best managed by the concomitant administration of atenolol. This *β*-adrenergic blocker is preferred, since it has the least inhibitory effect on the conversion of T4 to T3 [11]. The optimal dose of TH varies among individuals. Reduction of the serum TSH concentration to normal is a guide to therapy.

The 1st patient required levothyroxine 325 *μ*g/d and the 2nd 300 *μ*g/d. The dose of T3 or T4 required to achieve a beneficial effect is reported to be as high as 1000 *μ*g/d and 500 *μ*g/d, respectively. Triac is an acetic acid derivative of T4 and plays a minor role in normal thyroid physiology. However, it can be useful in the treatment of RTH, because it binds preferentially to the beta receptor [30]. Thus, adequate binding to certain mutated beta receptor can be achieved, without excessive stimulation of alpha receptors which predominate in the heart.

In conclusion, as the clinical presentation of RTH is atypical [31], the syndrome should be suspected whenever an individual presents with a high serum FT4 level, accompanied by a normal or even elevated TSH level. Despite the rarity of the condition, primary care physicians and endocrinologists should be alert when they encounter patients with these findings and a previous history of thyroidectomy receiving substitution therapy.

## Figures and Tables

**Table 1:** TSH, FT3, and FT4 levels and antithyroid medication in the first patient, before thyroidectomy.

TSH (*μ*UI/mL)	FT3 (pg/mL)	FT4 (ng/dL)	Medication
0.06	7.85	2.42	None: prescribed carbimazole 45 mg/d
5.16	4.8	1.92	Carbimazole 25 mg/d
0.94	3.83	2.39	Carbimazole 15 mg/d

Normal values: TSH = 0.4–4 *μ*UI/mL.

FT3 = 1.8–4.2 pg/mL.

FT4 = 0.8–1.9 ng/dL.

**Table 2:** TSH, FT3, and FT4 levels and levothyroxine therapy in both patients, after thyroidectomy.

	TSH (*μ*UI/mL)	FT3 (pg/mL)	FT4 (ng/dL)	Medication (levothyroxine)
1st patient	65.6	1.59	0.81	100 *μ*g/d
	22.7	3.51	2.4	200 *μ*g/d
	14.3	3.17	2.59	300 *μ*g/d
	5.5	4.3	2.91	325 *μ*g/d

2nd patient	32	4	1.87	150 *μ*g/d
	20.8	4.5	2.05	200
	10.3	4.3	3.16	250
	5.3	5	3.50	300

**Table 3:** Markers of metabolism on increasing doses of levothyroxine, in both patients.

	SHBG (nmol/L)	ALP (U/I)	Ferritin (ng/mL)	Cholesterol (mg/dL)	Triglycerides (mg/dL)	SGOT (U/I)	SGPT (U/I)	Medication (levothyroxine) (*μ*g/d)
1st patient	—	74	—	310	198	21	20	100
	18.7	81	—	—	—	20	18	200
	19.4	68	59.7	250	173	14	15	250
	21.7	80	46.5	247	165	12	15	300
	18	65	40	200	149	13	14	325

2nd patient	—	110	—	—	—	12	17	150
	8.17	125	21.1	190	55	14	16	200
	22.7	111	35.16	188	63	14	21	300

Normal values:

SHBG (sex hormone binding globulin): 18–114 nmol/L females, 13–71 nmol/L males.

ALP (alkaline phosphatase): 42–98 U/I.

Ferritin: 17–293 ng/mL males, 7–283 ng/mL females.

SGOT: 10–40 U/I.

SGPT: 10–35 U/I.
